# Improving the Accuracy and Safety of Allergy Documentation Through a Standardized Recording System: A Quality Improvement Project at Atbara Teaching Hospital

**DOI:** 10.7759/cureus.97358

**Published:** 2025-11-20

**Authors:** Mustafa Awad, Mohamed Elbashir, Aya Saad, Ithar Abdalla, Mohey Aldien Ahmed Elamin Elnour, Omer Alterrafy Mousa Suliman, Esra Elser Ibrahim Elhaj, Hager Elsir Sherfeldin Mohammed, Remaz Sayed Hassan, Ahmed Abdelaal, Riem Eltahir, Mohammed Osman Ahmed Osman, Moayad Mudawi, Mazin Elyas Alawad Mohammed, Ahmed Mohammed, Saad Adam Abdallah Abakar, Tayseer Salih Mohamed Salih, Eiman Yassir Musa Hussain, Aia Abdelsalam Elimam Ibrahim, Mohammad Alzain Adam

**Affiliations:** 1 Internal Medicine, Atbara Teaching Hospital, Atbara, SDN; 2 Medical Affairs, Al Rajhi Takaful Company, Riyadh, SAU; 3 General Practice, Atbara Teaching Hospital, Atbara, SDN; 4 Anesthesia and Critical Care, Atbara Teaching Hospital, Atbara, SDN; 5 Trauma and Orthopaedics, Atbara Teaching Hospital, Atbara, SDN; 6 Emergency Department, Atbara Teaching Hospital, Atbara, SDN; 7 Emergency Medicine, Atbara Teaching Hospital, Atbara, SDN; 8 Neonatology, Atbara Teaching Hospital, Atbara, SDN; 9 Anesthesia, ICU, and Perioperative Medicine, Atbara Teaching Hospital, Atbara, SDN; 10 Pulmonary Medicine, Aswan University Hospital, Aswan, EGY; 11 Internal Medicine, Sudan Medical Specialization Board, Khartoum, SDN

**Keywords:** allergy documentation, atbara teaching hospital, clinical audit, medical records, patient safety, quality improvement, sudan

## Abstract

Background: Incomplete allergy documentation is a major contributor to medication errors and adverse drug events. At Atbara Teaching Hospital, allergy information was inconsistently recorded, with no standardized format or visible alert system. This audit aimed to improve the completeness and accessibility of allergy documentation through the introduction of a structured sticker and brief staff training.

Methods: A two-cycle clinical audit was conducted in the Department of Internal Medicine at Atbara Teaching Hospital, Sudan, between August and October 2025. The first cycle reviewed 50 inpatient records to establish baseline compliance with international documentation standards. Following this, a standardized “Allergy Documentation Sticker” was designed and implemented, accompanied by short educational sessions for staff. The second cycle reviewed another 50 records post intervention. Data were analyzed using descriptive statistics and chi-square tests for significance.

Results: Baseline compliance with allergy documentation standards was poor. Fields such as allergen, reaction type, and severity were absent in all records (0/50, 0%). After introducing the sticker, compliance improved significantly to 50/50 (100%) for these parameters (p < 0.001). Documentation of "Any Known Allergy?" increased from 25/50 (50%) to 50/50 (100%) (p < 0.001), while doctor name and signature improved from 0/50 (0%) to 39/50 (78%) (p < 0.001). Minor improvements were also observed in demographic details, such as "unit" (38/50 (76%) → 45/50 (90%)). Overall, statistically significant gains were recorded across most fields, reflecting enhanced accuracy, accountability, and visibility of allergy records.

Conclusion: Introducing a structured allergy documentation sticker significantly improved the quality and completeness of allergy recording in a paper-based hospital setting. The intervention was low-cost, sustainable, and adaptable, aligning with global patient safety priorities. Regular audits, continued education, and progressive digital integration are recommended to sustain these improvements.

## Introduction

Accurate documentation of patient allergies, particularly drug allergies, is essential for ensuring medication safety and preventing avoidable adverse reactions. Incomplete or inaccurate allergy records can lead to serious prescribing errors, potentially resulting in morbidity, mortality, and increased healthcare costs worldwide [1]. Reports from the Agency for Healthcare Research and Quality indicate that drug allergy-related incidents account for a significant proportion of preventable adverse drug events, emphasizing the importance of structured allergy documentation systems [2].

However, numerous audits have shown that the completeness and accuracy of allergy documentation remain poor across many hospital settings. In a rural hospital audit, only 0.6% of patient records contained fully accurate and consistent allergy information across all clinical documents, despite nearly half of patients reporting allergies [3]. Similarly, an audit conducted in a National Health Service (NHS) hospital found that while allergy status was often recorded in some documents, inconsistencies and omissions across charts, clerking notes, and prescription sheets were common [4].

In developing countries, including those in low- and middle-income regions, the problem is further exacerbated by paper-based systems and a lack of standardized templates. A clinical audit from a tertiary care center in Pakistan revealed that allergy documentation in drug charts was initially present in only 22% of cases but improved to 78% following structured interventions involving education and audit feedback [5]. Such findings highlight that low-cost, context-appropriate interventions can significantly enhance documentation compliance and safety.

Interventional studies have demonstrated that combining staff education, structured forms, and regular audit cycles can lead to measurable improvements in the quality of allergy documentation. One study in a medical receiving unit reported that an educational campaign combined with feedback increased complete documentation rates across departments [6]. Moreover, recent analyses of electronic medical records have shown that even in advanced systems, a substantial proportion of antibiotic allergy labels are inaccurate or incomplete, reinforcing the need for better recording of allergens, reaction type, and severity [7].

At Atbara Teaching Hospital, internal audits revealed that while allergy status was often marked as “Yes” or “No,” detailed information, such as allergen name, reaction type, and severity, was rarely completed. To address this, a standardized allergy documentation sticker was developed and implemented across departments.

Study objectives

This audit aimed to assess the baseline compliance with allergy documentation standards across clinical departments at Atbara Teaching Hospital, to implement a structured allergy documentation sticker designed to improve recording accuracy and completeness, and to evaluate the impact of this intervention through a closed-loop, two-cycle audit process.

## Materials and methods

Study design and setting

This study followed a prospective, two-cycle clinical audit design, conducted between August and October 2025 at Atbara Teaching Hospital, Sudan. The hospital is a secondary-level referral institution providing internal medicine, surgical, and obstetric services to a wide catchment population within the River Nile State. The audit was conducted in the Department of Internal Medicine, where patient documentation is maintained exclusively on paper. Prior to this project, there was no structured or standardized format for allergy documentation, leading to frequent omissions and inconsistencies across admission notes, nursing charts, and prescription records.

Audit standards and criteria

Audit standards were derived from internationally recognized frameworks for medication safety, particularly the World Health Organization’s Global Patient Safety Challenge: Medication Without Harm and the National Institute for Health and Care Excellence (NICE) Clinical Guideline CG183 on drug allergy management [2,8]. These references emphasize that comprehensive allergy documentation is a fundamental element of safe prescribing and inter-professional communication.

Accordingly, five main criteria were used as audit standards. First, every admitted patient should be asked about allergy status during the initial assessment. Second, the allergy status must be clearly documented, indicating either the presence of a known allergy or explicitly stating “No Known Allergen (NKA)” to confirm that screening was completed. Third, in cases where allergies exist, the documentation should include detailed information about the allergen, the type of reaction, and its severity. Fourth, every entry should include the clinician’s name, signature, and date for accountability and traceability. Finally, allergy documentation must be clearly visible on the first page of the patient file to ensure accessibility across all clinical disciplines. These criteria collectively formed the benchmark for evaluating compliance in both audit cycles.

Data collection and sampling

The audit employed a total coverage sampling approach, where all eligible patient records within the defined audit period were reviewed. A total of 100 medical records were analyzed: 50 from the first audit cycle (pre-intervention) and 50 from the second cycle (post-intervention). Only adult inpatients admitted to the Internal Medicine Department were included, and records were required to contain completed admission forms, treatment charts, and discharge summaries.

Records were excluded if they belonged to pediatric or outpatient departments, if admission documentation was incomplete or missing essential clinical information, or if they originated from surgical or obstetric wards, as these departments maintain different documentation workflows. Data were collected using a structured audit proforma designed to reflect the fields of the standardized allergy documentation sticker. Each file was reviewed independently by two trained auditors, and discrepancies were resolved through consensus in consultation with a senior clinician. No personal identifiers were recorded at any stage to maintain confidentiality.

Intervention: The standardized allergy documentation sticker

Following completion of the first audit cycle, a standardized allergy documentation sticker was developed and introduced to enhance the accuracy and visibility of allergy recording. The sticker incorporated designated fields for patient identification, allergy inquiry, allergen name, reaction type, severity, age at diagnosis, additional notes, and clinician verification (name, signature, and date). The full layout and design of this sticker are presented in the Appendix, which illustrates the structured fields and embedded safety reminders used to promote complete and consistent documentation. This appendix visually complements the intervention description and supports reproducibility for other healthcare settings.

To support the implementation process, brief training sessions were organized for physicians, residents, and nursing staff, focusing on the clinical implications of inadequate allergy documentation and demonstrating correct completion of the sticker. Stickers were systematically affixed to the first page of each medical record, ensuring immediate visibility to all members of the healthcare team, including nurses and pharmacists. Compliance monitoring was conducted by departmental supervisors during the early implementation phase.

Data analysis

All collected data were entered into Microsoft Excel (version 2021; Microsoft Corporation, Redmond, WA) for organization and preliminary descriptive analysis. Each audit criterion was treated as a categorical variable (compliant/non-compliant), and compliance rates were calculated as percentages for both audit cycles. Improvement was measured as the absolute percentage change between the first and second cycles.

Comparative analysis between the two cycles was performed using the chi-square (χ²) test to determine whether observed differences in compliance proportions were statistically significant. For each test, the degrees of freedom (df) and effect size (Cramer’s V) were calculated to assess the magnitude of association. A p-value < 0.05 was considered statistically significant. All statistical computations were carried out using Microsoft Excel’s data analysis tools and cross-verified with IBM SPSS Statistics (version 26.0, IBM Corp., Armonk, NY) to ensure accuracy.

Ethical considerations

Ethical approval for this study was obtained from the Administrative and Ethical Committee of Atbara Teaching Hospital (IRB Number: At-QIP-4535; dated: September 20, 2025). Human patients were involved in this audit only through the review of their de-identified medical records. As the study involved retrospective analysis of anonymized inpatient files and posed no direct risk to patients, formal consent was not required. All data were handled in accordance with institutional confidentiality protocols and the ethical principles outlined in the Declaration of Helsinki. Access to patient files was restricted to authorized personnel, and the findings were used solely for quality improvement and educational purposes.

## Results

A total of 100 inpatient records were included (50 in cycle 1 and 50 in cycle 2), representing complete coverage of eligible internal medicine admissions during the study period. Baseline documentation of demographics was high (e.g., name and gender, each 50 (100%)), yet allergy critical fields were rarely completed: allergen, type of reaction, and severity were each 0 (0%) in cycle 1, while "Any known allergy?" appeared in only 25 (50%) records. Clinician verification elements were also absent (doctor name and doctor signature were each 0 (0%)), indicating weak accountability and fragmented processes.

After introducing the standardized allergy documentation sticker, cycle 2 showed transformational gains across all key medication-safety domains. Documentation of allergen, type of reaction, and severity increased from 0 (0%) to 50 (100%) (χ² = 100.0, df = 1, p < 0.001, Cramer’s V = 1.00), and "Any known allergy?" rose from 25 (50%) to 50 (100%) (χ² = 33.33, df = 1, p < 0.001, Cramer’s V = 0.58). Context-enriching fields also improved markedly: age of diagnosis from 0 (0%) to 49 (98%) (χ² = 96.08, df = 1, p < 0.001, Cramer’s V = 0.98) and additional notes from 0 (0%) to 43 (86%) (χ² = 75.44, df = 1, p < 0.001, Cramer’s V = 0.87), reflecting better clinical characterization and handover quality. Verification improved from 0 (0%) to 39 (78%) for both doctor name and doctor signature (χ² = 63.93, df = 1, p < 0.001, Cramer’s V = 0.80), signaling stronger ownership of documentation. Supporting identifiers (e.g., unit 38 (76%) → 45 (90%)) also trended upward (χ² = 3.47, df = 1, p = 0.062, Cramer’s V = 0.19), suggesting broader improvements in documentation culture catalyzed by the sticker and brief training.

These findings are detailed in Table 1, which presents counts with percentages, absolute improvement, and chi-square test statistics for each parameter. All major improvements demonstrated large effect sizes (Cramer’s V > 0.5), confirming a strong practical impact of the intervention.

**Table 1:** Comparison of allergy documentation between the first and second audit cycles at Atbara Teaching Hospital (August–October 2025). Comparison of allergy documentation parameters between the first and second audit cycles conducted at Atbara Teaching Hospital, Sudan (August–October 2025). Each cycle included 50 inpatient records (n = 50) from the Department of Internal Medicine. The table presents raw frequencies with percentages in parentheses, absolute percentage improvement, and chi-square (χ²) test results for significance of change. Degrees of freedom (df) = 1 for all comparisons; effect sizes were calculated using Cramer’s V. A p-value < 0.05 was considered statistically significant. Dash (—) indicates no statistical calculation possible due to complete compliance in both cycles. Data source: Quality Improvement Project on Allergy Documentation, Atbara Teaching Hospital (October 2025).

Item	First cycle (n = 50)	Second cycle (n = 50)	Improvement (%)	Chi-square (χ²)	p-value
Name	50 (100%)	50 (100%)	0	—	—
Age	49 (98%)	50 (100%)	+2	1.010	0.315
Hospital file number	49 (98%)	50 (100%)	+2	1.010	0.315
Gender	50 (100%)	50 (100%)	0	—	—
Department	46 (92%)	49 (98%)	+6	1.895	0.169
Unit	38 (76%)	45 (90%)	+14	3.473	0.062
Address	48 (96%)	50 (100%)	+4	2.041	0.153
Have you inquired about the patient’s allergy status?	50 (100%)	50 (100%)	0	—	—
Any known allergy?	25 (50%)	50 (100%)	+50	33.333	<0.001
Allergen (if none, write no known allergen)	0 (0%)	50 (100%)	+100	100.000	<0.001
Type of reaction	0 (0%)	50 (100%)	+100	100.000	<0.001
Severity	0 (0%)	50 (100%)	+100	100.000	<0.001
Age of diagnosis	0 (0%)	49 (98%)	+98	96.078	<0.001
Additional notes	0 (0%)	43 (86%)	+86	75.439	<0.001
Doctor signature	0 (0%)	39 (78%)	+78	63.934	<0.001
Doctor name	0 (0%)	39 (78%)	+78	63.934	<0.001
Date	49 (98%)	50 (100%)	+2	1.010	0.315

## Discussion

The results of this two-cycle audit demonstrate that a simple, low-cost, structured intervention (the allergy documentation sticker plus brief education) can significantly strengthen allergy recording practices in a resource-limited hospital environment. From zero to full compliance in critical fields such as allergen, type of reaction, and severity (0 → 50 (100%)) underscores the effectiveness of visible prompts and standardized templates in reducing variability and bridging the gap between knowledge and practice.

Several factors may explain the dramatic gains. First, the sticker functioned as a cognitive prompt; placing the allergy fields on the very first page of patient files forced clinicians to address them at the point of entry, reducing omission risk. This aligns with broader evidence showing that visual cues and templates can boost documentation compliance in clinical settings. For instance, a hospital-wide documentation improvement initiative used standard forms and workflow redesign to raise compliance rates across domains [9].

Second, the training sessions, although brief, likely raised awareness, created shared expectations, and reduced resistance to change. Studies of clinical documentation initiatives emphasize the importance of securing clinician buy-in, deploying “champions,” and embedding the intervention within existing workflows [10].

Third, by enforcing accountability (requiring the doctor's name and signature), the intervention created a system of traceability, which may have motivated better compliance. Documentation audits with personal feedback are documented strategies in nursing and medical quality improvement literature to maintain behavioral change [10].

Fourth, although this hospital uses paper records, the principles are congruent with electronic health record (EHR) improvement efforts: structured data entry, required fields, and alerting can reduce missingness in allergy modules. Indeed, EHR-based interventions have improved allergy documentation accuracy and timeliness when integrated into clinical decision support systems [11].

Our findings closely echo prior audits in diverse settings. In the UK, an audit of allergy documentation revealed major inconsistencies: while status was often recorded, accurate recording of the nature of allergy was seldom achieved in both medical clerking and drug charts [4]. Meanwhile, an ICU audit showed that enforcing mandatory allergy entry and involving pharmacists in reconciliation reduced prescribing before allergy status was known and improved record agreement between units [12].

In pediatric settings, efforts to improve penicillin allergy labels increased completeness from 20% to 64% over a year through education and form redesign, though still far from perfection [13]. Our audit achieved 100% in key fields within a shorter timeframe, suggesting that concentrated, context-tailored changes can yield more rapid impact in smaller units or departments.

Implications for practice and sustainability

These results have clear implications for patient safety. Complete allergy records reduce the risk of prescribing drugs that may trigger adverse reactions, improve inter-professional communication (nurses, pharmacists, physicians), and reinforce a culture of accountability. Over time, the hospital can institutionalize the sticker as a permanent component of the medical record and periodically audit compliance to sustain gains, closing the audit loop. This is in keeping with the core goal of clinical audit: to reduce the gap between standards and practice and embed continuous improvement [14].

To prevent regression and ensure the sustainability of these improvements, several strategies should be implemented. Regular audit and feedback cycles, ideally conducted on a quarterly basis, can help maintain staff accountability and reinforce adherence to documentation standards. In addition, refresher training and targeted reminders, particularly for newly joined staff, should be incorporated into departmental orientation programs to preserve awareness and consistency. Leadership endorsement is equally essential; integrating the allergy documentation sticker into official hospital policy and clinical governance frameworks would institutionalize the process and protect it from lapses during staff rotations or administrative changes. In the longer term, transitioning from the current paper-based system to electronic medical record templates with mandatory fields and automated alerts could further enhance the sustainability, accuracy, and accessibility of allergy data once digital infrastructure becomes available.

Strengths and limitations

This study demonstrated notable strengths, including its structured two-cycle audit design, cost-effective and context-appropriate intervention, and alignment with internationally recognized standards such as the WHO Global Patient Safety Challenge and NICE Guideline CG183. The use of standardized criteria, a reproducible sticker format, and comparative pre- and post-intervention analysis provides methodological rigor and external validity. The intervention itself (a simple, sustainable, and low-cost documentation sticker) proved highly feasible for replication in other resource-limited, paper-based healthcare settings. Ethical approval and transparent reporting of audit methodology further enhance the study’s credibility.

However, several limitations should be acknowledged. The relatively small sample size (n = 100) and single-center scope limit the generalizability of the findings. Additionally, the short follow-up period precluded assessment of the long-term sustainability of documentation improvements. The possibility of observer and Hawthorne effects cannot be excluded, as clinicians were aware of the ongoing audit. Furthermore, while the audit focused on documentation completeness, it did not verify the clinical accuracy of the reported allergy details. Despite these limitations, the study’s design remains robust for its intended quality improvement purpose and provides valuable insight into achievable, low-resource interventions. Future multicenter audits and longitudinal follow-ups are recommended to evaluate the sustained impact and broader applicability of this approach.

## Conclusions

This audit demonstrated that implementing a standardized allergy documentation sticker, supported by brief educational sessions, significantly improved the completeness and accuracy of allergy recording at Atbara Teaching Hospital. The observed improvements across all documentation parameters confirm that targeted, low-cost interventions can produce measurable and sustainable changes in clinical practice. While the findings are limited by the single-center scope and short follow-up duration, the intervention model offers a scalable, replicable approach suitable for similar resource-limited healthcare settings.

## Figures and Tables

**Figure 1:**
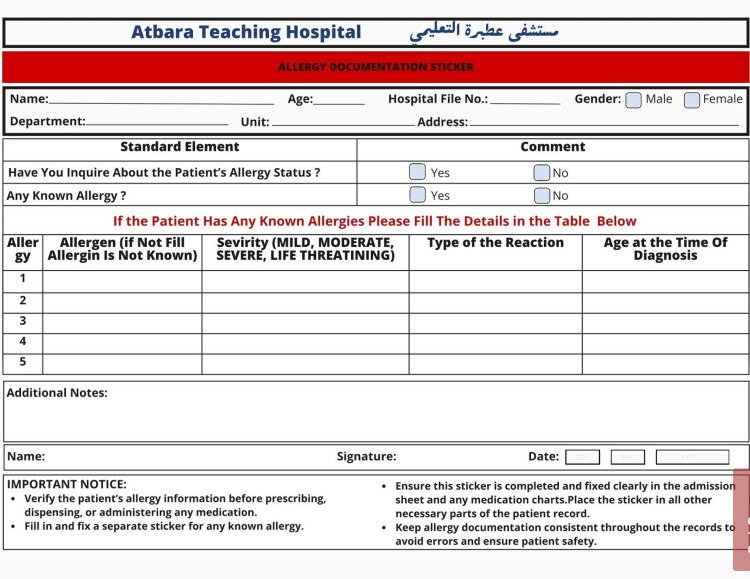
Newly designed allergy documentation sticker implemented at Atbara Teaching Hospital. The standardized allergy documentation sticker was developed for use in all inpatient medical records at Atbara Teaching Hospital. The form includes essential patient identifiers, a structured allergy inquiry section, and detailed fields for recording allergen type, reaction severity, and age at diagnosis. It also contains safety reminders for clinicians regarding verification of allergy status prior to prescribing or administering medications. The sticker is affixed to patient files, admission sheets, and medication charts to enhance visibility, minimize documentation errors, and improve patient safety.
